# Isolation, Identification, and Characterization of Polystyrene-Degrading Bacteria From the Gut of *Galleria Mellonella* (Lepidoptera: Pyralidae) Larvae

**DOI:** 10.3389/fbioe.2021.736062

**Published:** 2021-08-18

**Authors:** Shan Jiang, Tingting Su, Jingjing Zhao, Zhanyong Wang

**Affiliations:** ^1^ School of Petrochemical Engineering, Liaoning Petrochemical University, Fushun, China; ^2^ Department of Biotechnology, College of Bioscience and Biotechnology, Shenyang Agricultural University, Shenyang, China

**Keywords:** galleria mellonella, gut microbiome, polystyrene, biodegradation, massilia sp.

## Abstract

Polystyrene (PS) is a widely used petroleum-based plastic, that pollutes the environment because it is difficult to degrade. In this study, a PS degrading bacterium identified as *Massilia* sp. FS1903 was successfully isolated from the gut of *Galleria mellonella* (Lepidoptera: Pyralidae) larvae that were fed with PS foam. Scanning electron microscopy and X-ray energy dispersive spectrometry showed that the structure and morphology of the PS film was destroyed by FS 1903, and that more oxygen appeared on the degraded PS film. A water contact angle assay verified the chemical change of the PS film from initially hydrophobic to hydrophilic after degradation. X-ray photoelectron spectroscopy further demonstrated that more oxygen-containing functional groups were generated during PS degradation. After 30 days of bacterial stain incubation with 0.15 g PS, 80 ml MSM, 30°C and PS of Mn 64400 and Mw 144400 Da, the weight of the PS film significantly decreased, with 12.97 ± 1.05% weight loss. This amount of degradation exceeds or is comparable to that previously reported for other species of bacteria reported to degrade PS. These results show that *Massilia* sp. FS1903 can potentially be used to degrade PS waste.

## Introduction

Petroleum-based plastics are artificial organic polymers, obtained from natural gas or oil and used for a variety of civil and industrial applications (Suman et al., 2020). According to new data, the global output of petroleum-based plastic materials has increased to approximately 359 million tons per year, the demand for them account for approximately 80% of the total plastics used (PlasticsEurope, 2019). Polyethylene, polypropylene, polyvinyl chloride, polystyrene (PS), polyurethane and polyethylene terephthalate plastics are the main types of petroleum-based plastics (Wei and Zimmermann, 2017). Because of their profound stability and the lack of suitable degradation methods, these plastics gradually accumulate in the environment, leading to a sharp increase in environmental contamination and substantial waste (Barnes et al., 2009). Researchers have even detected plastic fragments in deep sea sediments at a depth of 5,000 m. These forms of plastic are absorbed by marine organisms, which cause serious health problems for these organisms and may also affect human health (Rochman et al., 2015; Zhang et al., 2020).

PS, as the third most important petroleum-based plastic, is used for packaging containers, disposable cups and insulating materials, and comprises about 7% of the total amount of plastics produced (Ho et al., 2018; Urbanek et al., 2020). Because of its long persistence in the environment, PS wastes biodegradation efficiency is extremely low in natural ecosystems, and cased serious environmental pollution (Wilkes and Aristilde, 2017). Therefore, researchers around the world are exploring various PS high-efficiency degradation pathways without secondary pollution. Recently, many researchers began to investigate the biodegradation potential of intestinal microorganisms, especially in insect larvae with chewing mouthparts. Yang et al. (2015) isolated a PS-degrading bacterial strain *Exiguobacterium* sp. YT2 from the gut of *Tenebrio molitor*. This research showed that over a 30-days incubation period, YT2 formed a biofilm covering PS, resulting in obvious pits and cavities on the PS film surfaces. Over a 60-days incubation period, a suspension culture of strain YT2 degraded 7.4 ± 0.4% of the PS pieces. Another group of researchers subsequently showed that a previously untested strain of *T. molitor* larvae could degrade PS (Yang et al., 2017). Moreover, Peng et al. (2019) compared the PS degradation capability of *T. molitor* and *Tenebrio obscurus*. The results demonstrated that the ability of the *T. obscurus* gut to degrade PS is higher than that of *T. molitor* and that the PS consumption rate is also greater. After 31 days of feeding, the molecular weight of residual PS in the frass of *T. obscurus* decreased by 26.03%. Other insect species also have the same abilities as mealworms to degrade PS. Yang et al. (2020) showed that *Zophobas atratus* can use PS foam as their only food source, and various techniques have been used to demonstrate that the depolymerization of long-chain PS molecules and the subsequent formation of low-molecular-weight products occur in the larval gut. On the basis of this research, *Pseudomonas* sp., which can degrade PS, was isolated from the gut of *Z. atratus* larvae by Kim et al. (2020). Likewise, Wang et al. (2020) observed that *Tribolium castaneum* larvae can chew and eat PS foam, and finally successfully isolated a bacterial strain, identified as *Acinetobacter* sp. AnTc-1, from the gut of these larvae. Woo et al. (2020) reported PS biodegradation by the larvae of *Plesiophthalmus davidis* and isolated a bacterial strain *Serratia* sp. WSW (KCTC 82146) from their gut flora.

*Galleria mellonella* (Lepidoptera: Pyralidae) is a common agricultural pest that destroys the structure of honeycombs and are generally considered to be of no benefit to human beings; however, it has been discovered that *G. mellonella* larvae can eat and biodegrade polyethylene and PS (Lou et al., 2020). According to previous reports, this phenomenon is likely related to the gut microorganisms of these insects (Yang et al., 2015; Peng et al., 2019; Yang et al., 2020). In addition, some microbes from the gut of *G. mellonella* larvae related to the degradation of PE has been confirmed, and a PE degrading strain *Enterobacter* sp. D1 has been successfully screened (Bombelli et al., 2017; Ren et al., 2019; Cassone et al., 2020). However, there are few studies on PS degrading bacteria from the gut of *G. mellonella* larvae. In this study, a PS degrading bacterium was successfully isolated from the larval gut and identified by phylogenetic analysis combined with physiological and biochemical indicators. To determine the extent of PS degradation by the bacterium, the physicochemical properties of the degraded PS film were studied by a scanning electron microscope (SEM), X-ray energy dispersive spectrometer (EDS), water contact angle (WCA), and X-ray photoelectron spectroscopy (XPS) analyses.

## Materials and Methods

### Experimental Materials

*G. mellonella* larvae were purchased from Huiyude Co. (Tianjin, China). PS foam board was obtained from Nannan Building Materials Co. (Zhejiang, China) and contained polystyrene purity over 98%. The number-average molecular weight (Mn) and weight-average molecular weight (Mw) of the PS were 64400 and 144,400 Da, respectively, as measured by gel permeation chromatography. The PS film for microbial degradation was prepared using the previous method (Yang et al., 2015) and fine-tuned for this study: the foam PS was dissolved in dichloromethane (0.03 g/ml) and then the solution was spread on a glass plate, after 5 h, the resulting film was removed from the glass plate and immobilized in a fume hood for 3 days at room temperature. The film was then rinsed with de-ionized water and dried before use. The thickness of the prepared film was approximately 0.02 mm.

The composition of the mineral salts medium (MSM, pH = 7.01) was as follows: 4.54 g/L KH_2_ PO_4_, 11.94 g/L Na_2_HPO_4_·12H_2_O, 1.0 g/L NH_4_Cl, 0.5 g/L MgSO_4_, 0.005 g/L CaCl_2_, 0.002 g/L FeSO_4_, 0.001 g/L MnSO_4_ and 0.002 g/L ZnSO_4_. The Luria-Bertaini (LB) medium was prepared by dissolving 10 g NaCl, 10 g tryptone, and 5 g yeast extract in 1 L deionized water. 15 g agar was added to prepare the LB agar medium. 0.9 g NaCl was dissolved in 100 ml deionized water to prepare saline. All media buffers and solutions were subjected to high-pressure steam sterilization (121°C, 103.4 kPa, 20 min). All other chemical reagents used in this study were analytical reagent grade and obtained from commercial sources.

### Screening of Strains for PS Degradation Ability

Larvae (n = 200) were fed with PS foam for 21 days, and then 10 larvae were collected. After sterilization, the larvae were dissected and then the intestinal tissue was placed into a 1.5 ml centrifuge tube containing 1 ml of normal saline, and then shaken on a vortex mixer for 5 min. A pure intestinal cell suspension was obtained and used as a bacterial inoculum to enrich PS-degrading bacteria. A 1 ml aliquot of the suspension was transferred into a 250-ml Erlenmeyer flask with 80 ml MSM and 0.15 g PS film (1 × 3 mm), which was shaken on a rotary shaker (120 rpm) at room temperature. After 60 days, the remaining PS film was removed, and the enrichment culture was dispersed on LB agar plates. After culturing for 24 h at room temperature, colonies were picked and then spread on fresh LB agar plates until a pure colony was finally obtained according to the standard methodology of bacterial isolation (Yang et al., 2015). After the pure bacterial isolates were grown in liquid LB medium for 12 h, the cells were collected by centrifugation (10000 rpm) and then washed with sterile water to remove residual medium. This step was repeated until there were no remaining nutrients. Next, the collected cells were resuspended in sterile water and then diluted 100 times to obtain a cell suspension (Yang et al., 2015). The cell suspension (0.4 ml) was distributed evenly on the surface of a MSM plate, which was then covered with PS film. Two control groups were set up. One was only inoculated with bacterial culture, while the other was only covered with PS film. All plates were cultured in triplicate at 30°C for 30 days. Place an open Petri dish with distilled water in the incubator to prevent the plates from drying. Changes in the film and bacterial growth were regularly observed. The remaining larvae were refrigerated at 4°C after pupation for subsequent use.

### Sequencing and Phylogenetic Analysis

**FIGURE 1:**
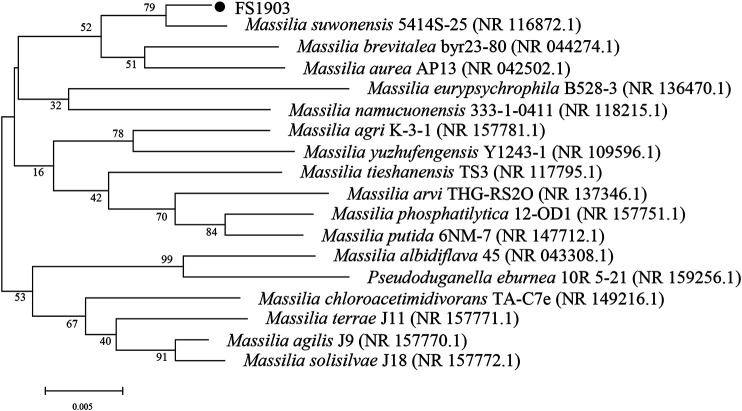
Neighbor-joining phylogenetic tree of strain FS1903 based on the 16S rRNA gene.

Genomic DNA used for 16S rDNA amplification was extracted from the cells during the logarithmic growth stage using the TIANamp Bacteria DNA Kit (Tiangen Biotech Co., Ltd., Beijing, China). Next, PCR amplification and agarose gel electrophoresis were performed to verify successful extraction. The gene was amplified using the universal primers 27-F (5′-AGA​GTT​TGA​TCC​TGG​CTC​AG-3′) and 1492-R (5′-GGT​TAC​CTT​GTT​ACG​ACT​T-3′). Amplicons in the gel were recovered using TIANquick Midi Purification Kit (Tiangen Biotech Co., Ltd., Beijing, China), and then sequenced using a kit from Sangon Biotech Co., Ltd. (Shanghai, China). The obtained sequences were aligned with known organisms in the GenBank database using the Basic Local Alignment Search Tool (BLAST) created by the National Center for Biotechnology Information (NCBI, Bethesda, MD, United States). A phylogenetic tree was constructed with MEGA5.0 software using the neighbor-joining method. Bootstraps of the supporting tree branches were constructed with 1,000 replications and the default settings. A total of 17 16S rRNA sequences were used for the phylogenetic analyses, and the accession numbers of these sequences are shown in Figure 1.

### Determination of Microbial Physiological and Biochemical Indicators

The determination of microbial physiological and biochemical indicators was carried out using the method of bacterial identification in the “Berger’s Bacterial Identification Manual” (eighth edition) and Identification Manual of Common Bacteria System (Buchanna & Gibbons 1984; Dong & Cai 2001).

### Performance Analysis of the Surface of the PS Film

An SEM (SU8010, Hitachi, Tokyo, Japan) was used to inspect the biofilm that formed on the PS film and the holes in the PS film after degradation. To observe the presence and growth of microorganisms, the PS films that covered the agar plates were removed after 30 days, fixed with 2.5% glutaraldehyde for 3 h and dehydrated with an ethanol gradient (30, 50, 70, 90 and 100%) for 15 min (Wang et al., 2020). To detect changes in the physical and chemical properties of the PS film, the films were immersed in a 2% (w/v) SDS aqueous solution for 4 h, and then washed with de-ionized water to completely remove the biofilm from the surface. The PS film from the uninoculated control was also treated in the same way (Sivan et al., 2006). To verify changes in the elemental composition of the PS film surface during the degradation process, a module connected to the SEM (EDS (SU8010, Hitachi, Tokyo, Japan) was used to evaluate the differences in the composition of the carbon and oxygen elements before and after degradation.

The WCA (DSA100, KRUSS, Hamburg, Germany) was used to analyze changes in the hydrophilicity of the PS film surface. The contact angle was measured under static conditions at room temperature. The PS film was washed three times with SDS solution and then de-ionized water, and the contact angle measurement was estimated five times.

To investigate degradation and determine the variations of functional groups on the surface of the PS film, XPS (ESCALAB Xi+, Thermo Fisher, Massachusetts, United States) was used to measure the binding energy. The PS film (1 cm × 1 cm) was fixed on a carbon ribbon and measured within the energy range of 2 + P−300eV, C1s.

### Biodegradation Assay

After overnight incubation in 4 ml liquid LB medium, bacteria were collected by centrifugation for 10 min at 10,000 rpm, and then washed three times with 4 ml of saline. PS film (1 cm × 3 cm, 0.15 g) was added into a 250-ml Erlenmeyer flask containing 80 ml MSM. A 1 ml suspension of the bacterial culture was added into the flask, which was then incubated on an orbital shaker (150 rpm) at 30°C. This method was a slight modification of the following reported scheme (Wang et al., 2020) because the PS powder was replaced with a film; the reaction system volume was doubled; the temperature and speed were fine-tuned higher; and the reaction time was 30 days. The PS film incubated in MSM without bacteria served as a control. The weight loss assay was replicated three times. After a 30-days incubation, the PS films were harvested (Mor and Sivan, 2008).

The weight loss of the PS film was calculated using the following formula:

Percentage of weight loss = 100% × (Initial PS weight-Final PS weight)/Initial PS weight

### Statistical Analysis

Statistical ANOVAs were performed using SPSS 20.0 (SPSS Inc., Chicago, United States) to evaluate the differences in contact angle changes and weight loss produced by bacteria. Pairwise comparisons were analyzed with the student’s t-test, as all date were normally distributed. All error values are reported as the mean value ±standard deviation.

## Results

### Feeding Behavior of *G. mellonella* Larvae on PS Foam

**FIGURE 2:**
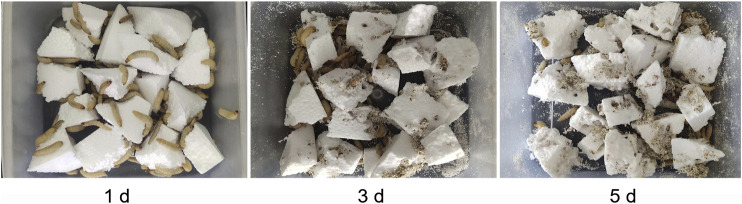
Holes formed by *G. mellonella* larvae after feeding on PS foam for 1, 3 and 5 days.

*G. mellonella* larvae were fed with PS for 21 days. By the third day of feeding, the PS foam block was already full of holes and a small amount of block was gnawed and attached to the culture container. By the fifth day, the whole culture container was covered with PS debris (Figure 2). After consuming the PS foam diet for 21 days, the larva appeared to be normal and they produced a normal amount of silk. These results suggested that the PS diet did not harm the larvae over a short-term feeding period.

### Preliminary Screening of Strains With PS Degradation Ability

**FIGURE 3:**
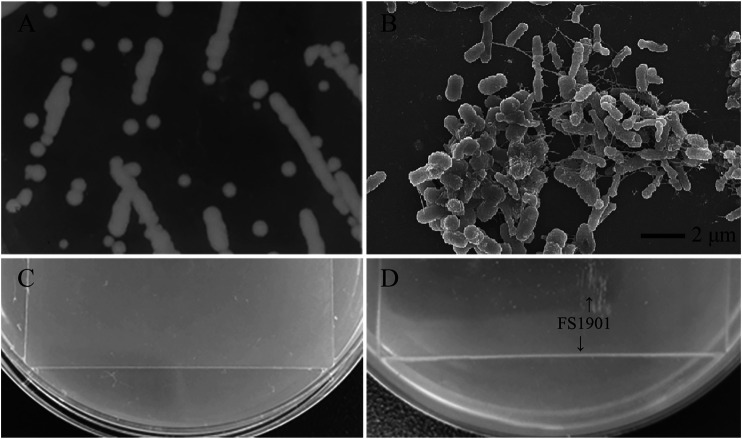
Colonies of FS1903 on LB agar plates **(A)**; SEM of FS 1903 **(B)**; the control **(C)**; and the colonies and lawn of FS1903 on the PS film on the MSM plate **(D)**.

After 2 months enrichment culture, when compared with the control group, a turbid liquid was observed in the experimental group, indicating that some intestinal bacteria with the ability to degrade and use PS as a nutrition source may have been enriched. After cultivation and purification, a pure colony named as FS1903was finally obtained. When cultivated on solid LB medium, FS1903 were round with clear edges and a slightly yellowish color (Figure 3A). The shape of the bacterial cells examined with an SEM. The results showed that FS1903 cells are an elliptical rod shape approximately 1.5 μm long and 0.5 μm in diameter (Figure 3B). To verify the PS degrading ability of FS 1903, a PS film (50 mm × 50 mm) was supplied as the sole carbon source on top of an MSM plate that was spread with the cell suspension. After 30 days cultivation, when compared with control group (Figure 3C), some small colonies were observed under the PS film and a linear bacterial lawn appeared at the edge of the film in the experimental group (Figure 3D). This provides evidence that FS1903 isolated from the gut of *G. mellonella* larvae can degrade PS.

### Physiological and Biochemical Test of the Tested Strain

The result of strain culturing showed that the tested strain is short rod-shaped bacterium, is motile by a terminal flagellum; forms smooth, round yellow-white colonies on LB medium; and optimum growth at 30°C and pH 7.0. Physiological and biochemical characteristics of the strain were list at Table 1. According to the physiological and biochemical characteristics results, the strain is similar to the microorganisms of the genus *Massilia* (Shen et al., 2013; Orthova et al., 2015). In addition, bacteria of this family are often isolated from the rumen or the large intestine of humans and animals (Kämpfer et al., 2012), this is also in line with our research.

**TABLE 1:** Physiological and biochemical characteristics of strain FS1903.

Characteristic	FS1903
Maltose	+
Glucose	+
Lactose	-
Rhamnose	+
Sucrose	+
Arabinose	+
Starch	+
Esculin	+
Sorbitol	-
Inositol	+
l-tyrosine hydrolysis	-
V-P (2d)	-
V-P (6d)	-
Contact enzyme	+
Propionate	+
gelatin liquefaction	+

### Sequencing and Phylogenetic Tree Construction of FS1903

A phylogenetic tree of FS1903 and other *Massilia* bacteria was constructed based on the 16S rRNA gene (Figure 1). According to the phylogenetic tree, FS1903 and *Massilia suwonensis* 5414s-25 were on the same branch and closely related with high sequence similarity of 79%. Combined with the physiological and biochemical test, tested strain can be identified as the genus *Massilia*, and then the strain was named *Massilia* sp. FS 1903. The 16S rRNA gene sequence of this bacterium was deposited in the GenBank database under accession number MW138062.

### Observation of the Biofilm Formed on the PS Film Surface

**FIGURE 4:**
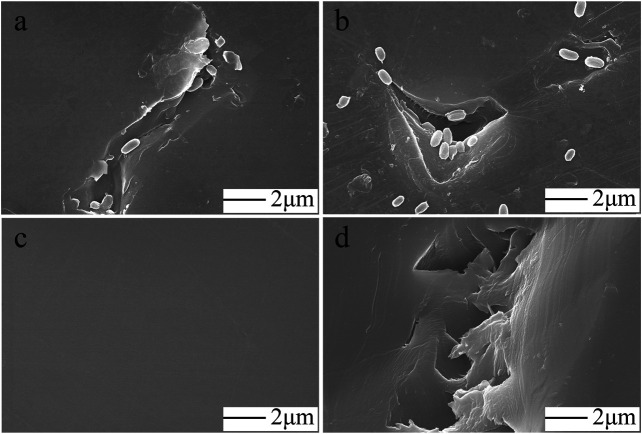
SEM observations. FS1903 cells on the degraded PS film **(A, B)**, control **(C)** and cavities and pits on the surface caused by incubation with FS 1903 **(D)**.

The biofilm was scanned with an SEM to more closely examine the effects of the colonization of the PS film surface by FS 1903. After 30 days of cultivation, the PS film surface was examined before and after the microorganisms were completely washed off, and the results showed that the PS film was damaged by the bacteria (Figure 4A,B). In contrast, the surface of the uncultured control was smooth (Figure 4C) without any defects. These observations revealed that the bacteria caused pits and cavities on the film surface. The maximum width of a typical cavity was approximately 4 μm (Figure 4D).

### Analysis of the Composition of the PS Film Surface

**FIGURE 5:**
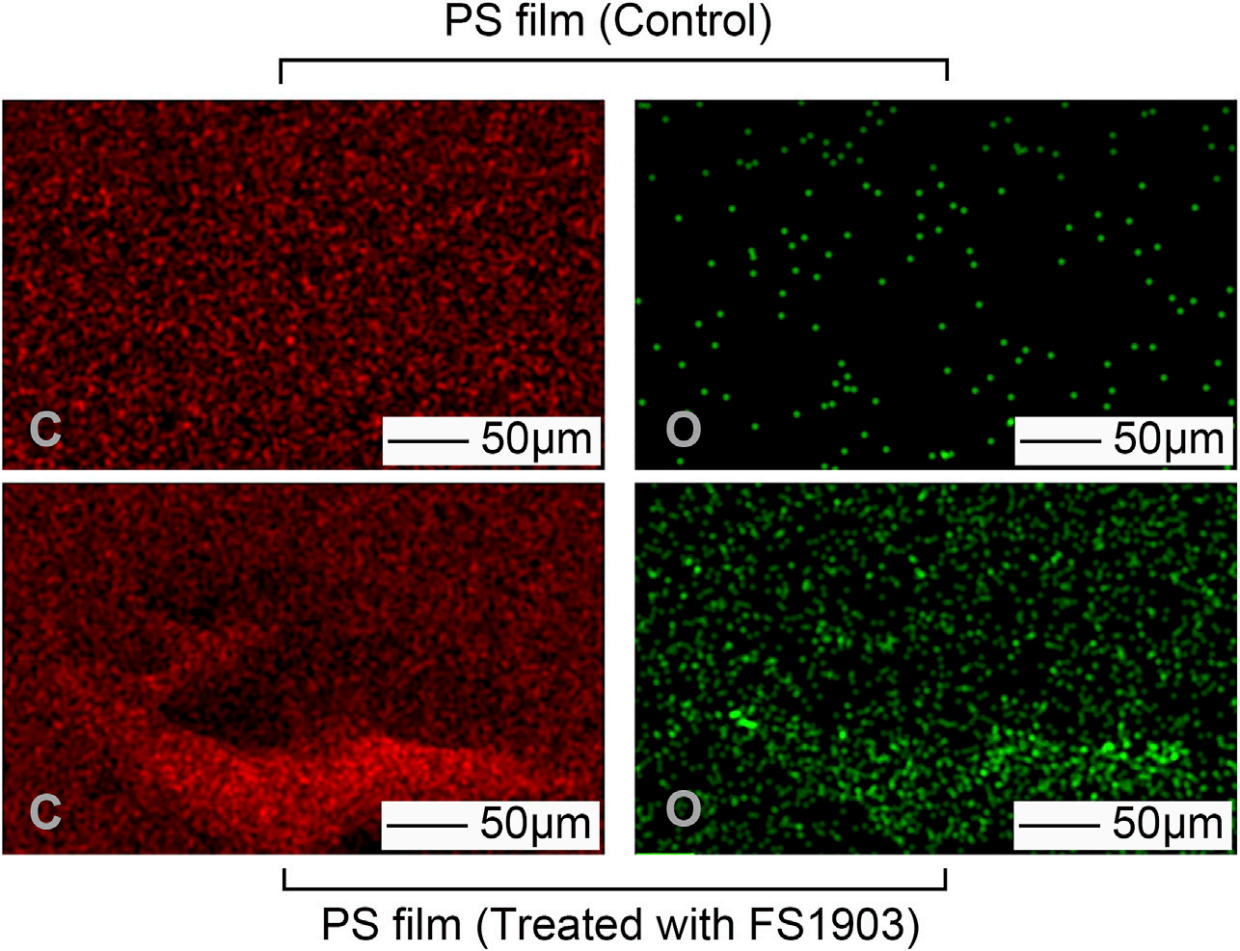
Changes in atomic composition of the PS film surface after treatment with FS 1903. Note: “C” represents carbon and “O” represents oxygen.

The carbon and oxygen composition on the PS surface where the bacteria grew was analyzed by EDS to determine the effects of the bacteria on the composition of the film. There were no obvious differences in the number of carbon atoms between the PS in the control group and the experimental group (Figure 5). However, more oxygen atoms were detected in the experimental group, which shows that oxidization occurred.

**FIGURE 6:**
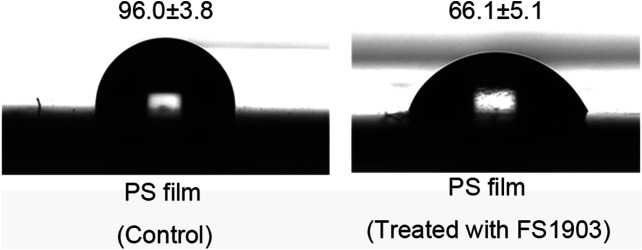
WCA of PS film without or with inoculation by strain FS1903.

Oxidation was confirmed by measuring changes in the contact angle of water droplets on the PS surface, which is indicative of the surface hydrophobicity of the PS film. The results show that the contact angle of the experimental group was 66.1 ± 5.1^o^, while that of the control group was 96.0 ± 3.8^o^ (Figure 6). Compare to the control, the contact angle of the experimental group was significantly decreased (*p* < 0.01). This decrease in the contact angle shows that the surface tension of water decreased, because of the insertion of oxygen on the surface of PS during the oxidation process. Oxidation during PS degradation converts hydrophobic regions to hydrophilic ones, thereby changing the chemical properties of the PS surface (Gu, 2003). FS1903 colonization reduced the hydrophobicity of the PS film and at the same time the film was damaged, the decreased hydrophobicity reduced the resistance to bacterial cells to subsequent PS degradation.

**FIGURE 7:**
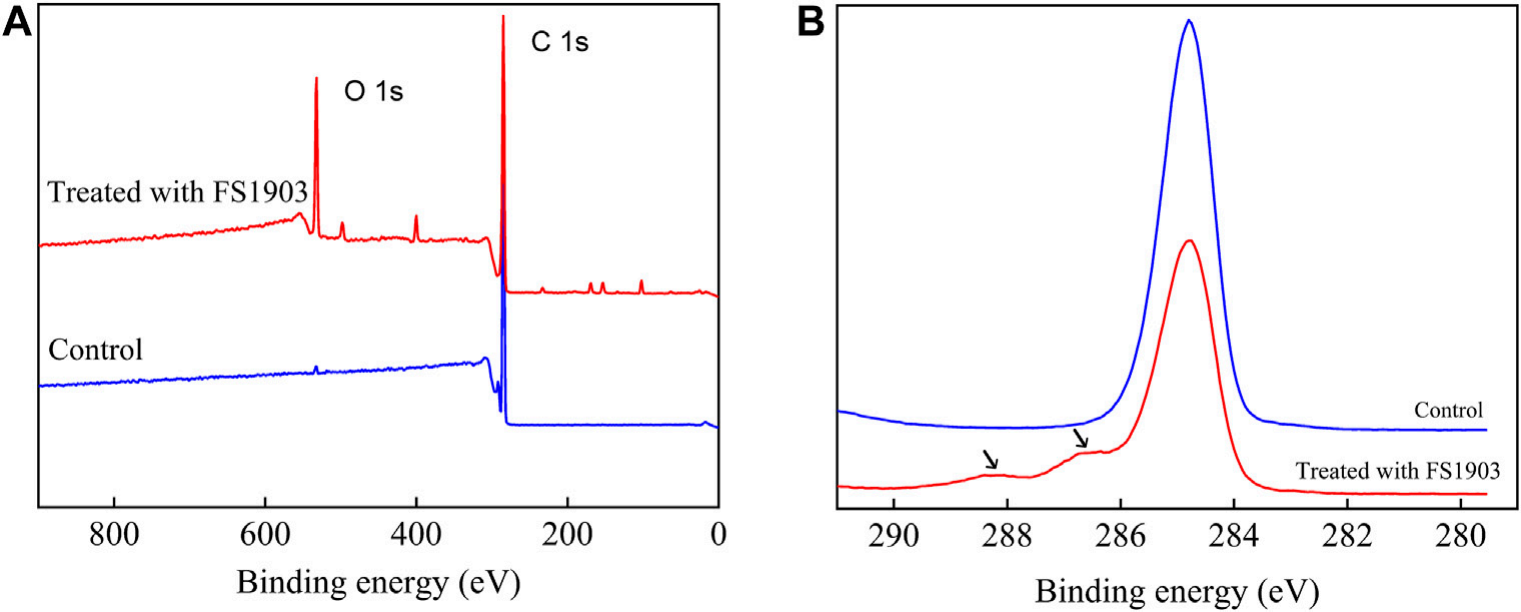
XPS scanning **(A)** and C1s spectra **(B)** of the control and the residual PS films inoculated with FS1903.

XPS was used to analyze changes in the surface chemical composition and functional groups. Figure 7 shows the comparison (0–900 eV) of the XPS scanning spectra of the PS film for the inoculated experimental and non-inoculated control groups. The control group had only surface carbon at peak of 284.8eV, while the PS inoculated with strain FS 1903 except 284.8eV had another obvious peak at 532.3 eV that represented the amount of surface oxygen (Figure 7A). The XPS spectra of C1s on the PS film surface inoculated with strain FS1903 versus the control was compared (Figure 7B), and it showed that culturing with the FS1903 caused a notable decrease of the C-C group (284.8eV). Meanwhile, compare to the control group with one peak, the PS inoculated with strain FS 1903 had another peak at 286.5 eV, which was assigned to the C−O group. This implies that part of the C−C groups in the PS was oxidized to alcohol and carboxylic-acid-like compounds (Shang et al., 2003). In addition, on the PS film surface inoculated with strain FS 1903, another new peak appeared at 288 eV, which was assigned to the −C=O group. These observations all demonstrate a transition from a C−C bond to −C=O and C−O bonds during the degradation process. Furthermore, these results indicate that strain FS1903 is capable of attacking or oxidizing the PS structure to produce more polar derivatives. This change in binding energy confirms that oxidation occurs during FS1903-mediated degradation. The results are in accordance with previous work (Yang et al., 2015; Kim et al., 2020).

### Biodegradation Assay

To confirm the degradation ability of strain FS 1903, we calculated the weight loss after infestation with the bacteria. After cultivation with the bacteria for 30 days, the weight loss of PS treated with strain FS1903 was 12.97 ± 1.05%, which was significantly decreased compared with the control group (*p* < 0.01). Furthermore, FS1903 degraded the PS without being supplied with nutritional supplements, such as yeast extract or gelatin.

## Discussion

According to previous literature (Yang et al., 2015; Yang et al., 2018; Peng et al., 2019), using antibiotics to inhibit gut microorganisms of certain insects that can feed on plastic directly leads to the loss of the insects’ ability to degrade plastic. At the same time, microorganisms that can degrade plastic polyethylene or PS from insect intestinal microbiomes were isolated (Yang et al., 2015; Kim et al., 2020; Wang et al., 2020). Therefore, it is clear that plastic-degrading bacteria do exist in the gut of some insects. Since the *G. mellonella* larvae have the ability to eat polystyrene and still grow normally, we preliminarily speculated that there may be microorganisms that can digest PS in the gut of these larvae. Our results confirmed this hypothesis and screened a strain of PS degrading bacteria.

The most notable and fundamental characteristic of bacteria-mediated plastic degradation processes is the formation of a biofilm on the plastic surface (Kim et al., 2020). Biofilms form on a substrate surface and can further penetrate into the materials to erode and deteriorate them (Chauhan et al., 2018; Liu et al., 2020). Adherence of bacterial cells to the polymer surface is the first and most basic step of subsequent biodegradation (Esmaeili et al., 2013). In this study, the bacterial strain FS 1903 can form a biofilm on PS film. After the biofilm is formed, PS is oxidized. Oxidation, transform the PS surface from hydrophobic to hydrophilic, is a key step in PS biodegradation (Tribedi and Sil, 2014). The oxidation in the experimental group is attributed to the functional groups (such as hydroxyl or carbonyl groups) formed via β-oxidation (Bode et al., 2000). This phenomenon caused by an enzyme or a variety of enzymes secreted by bacterial cells attaching to PS film that promotes the degradation and oxidation of PS (Mohanan et al., 2020). According to the analysis results of the composition, contact angle, and functional groups of the PS film surface, oxidation was produced in the PS films treated by FS 1903. These results confirmed that FS1903 can destroy the physical integrity of a PS film and degrade it.

Although the physics and chemistry changes in the PS inoculated with the bacteria demonstrate the degradation by FS 1903, the most direct evidence of PS biodegradation is its weight loss (Yang et al., 2015; Kim et al., 2020; Wang et al., 2020). *Rhodococcus* C208 and *Exiguobacterium* sp. YT2 have been previously shown to reduce the PS weight by 0.8% in 56 days and 7.4% in 60 days, respectively (Mor and Sivan, 2008; Yang et al., 2017). *Acinetobacter* sp. AnTc-1 can degrade PS powder, with a weight loss of 12.14 ± 1.4% after 60 days of cultivation (Wang et al., 2020). Compared to these other bacterial strains, FS1903 produced a higher weight loss in less time and demonstrated a comparable potential in degrading PS. However, the weight loss is very low compared to the direct consumption of PS by insect larvae. Previous researches have reported that plastic-degrading microorganisms demonstrate expedited degradation within the insect gut environment (Przemieniecki et al., 2020; Brandon et al., 2021). This phenomenon implies that the insect host may play a role in the plastic biodegradation process. *G. mellonella* larvae have been shown that they retain the capacity to metabolize polyethylene when their gut microbiome activity is suppressed (Kong et al., 2019). In addition, Brandon et al. (2021) and Tsochatzis et al. (2021) provide evidence that insect larvae secrete emulsifying factors or enzymes that mediate plastic biodegradation. These studies indicate that using insect larvae to degrade plastic is a complex system in which the larvae host and its gut microbiome collaborate to work. This may be the reason for the low weight loss of PS degradation by FS1903 only. Therefore, further identification key functional enzymes from larvae host and gut microbiome related to the depolymerization and biodegradation of PS is needed.

## Conclusion

This is the first study to identify a PS-degrading strain of bacteria isolated from the gut of *G. mellonella* larvae. The degradation ability of this strain FS1903 was comparable or better than any other bacterial strain previously identified. Optimization of conditions to simulate the intestinal environment and improve the degradation efficiency of these bacteria *in vitro* is currently being investigated in our laboratory. In addition, further work is needed to determine if the larvae have the ability to degrade other common plastics (such as polyethylene, polypropylene, polyvinyl chloride and polyethylene terephthalate), and to identify the mechanisms and pathways involved in this biodegradation.

## Data Availability

The datasets presented in this study can be found in online repositories. The names of the repository/repositories and accession number(s) can be found below: https://www.ncbi.nlm.nih.gov/nuccore/MW138062.1.
